# Does antiretroviral treatment increase the infectiousness of smear-positive pulmonary tuberculosis?

**DOI:** 10.5588/ijtld.17.0162

**Published:** 2017-11-01

**Authors:** P. Y. Khan, A. C. Crampin, T. Mzembe, O. Koole, K. L. Fielding, K. Kranzer, J. R. Glynn

**Affiliations:** * Department of Infectious Disease Epidemiology, London School of Hygiene & Tropical Medicine, London, UK; † Karonga Prevention Study, Chilumba, Malawi; ‡ National and Supranational Mycobacterium Reference Laboratory, Forschungszentrum Borstel, Germany

**Keywords:** *M. tuberculosis* infection, infectiousness, tuberculosis, antiretroviral treatment, HIV

## Abstract

**BACKGROUND:**

Understanding of the effects of human immunodeficiency virus (HIV) infection and antiretroviral treatment (ART) on Mycobacterium tuberculosis transmission dynamics remains limited. We undertook a cross-sectional study among household contacts of smear-positive pulmonary tuberculosis (TB) cases to assess the effect of established ART on the infectiousness of TB.

**METHOD:**

Prevalence of tuberculin skin test (TST) positivity was compared between contacts of index cases aged 2–10 years who were HIV-negative, HIV-positive but not on ART, on ART for <1 year and on ART for ⩾1 year. Random-effects logistic regression was used to take into account clustering within households.

**RESULTS:**

Prevalence of M. tuberculosis infection in contacts of HIV-negative patients, HIV-positive patients on ART ⩾1 year and HIV-positive patients not on ART/on ART <1 year index cases was respectively 44%, 21% and 22%. Compared to contacts of HIV-positive index cases not on ARTor recently started on ART, the odds of TST positivity was similar in contacts of HIV-positive index cases on ART ⩾1 year (adjusted OR [aOR] 1.0, 95%CI 0.3–3.7). The odds were 2.9 times higher in child contacts of HIV-negative index cases (aOR 2.9, 95%CI 1.0–8.2).

**CONCLUSIONS:**

We found no evidence that established ART increased the infectiousness of smear-positive, HIV-positive index cases.

## Abstract (FR)

**CONTEXTE:**

La compréhension des effets du virus de l'immunodéficience humaine (VIH) et du traitement antirétroviral (ART) sur les dynamiques de transmission de Mycobacterium tuberculosis reste limitée. Nous avons entrepris une étude transversale auprès des contacts domiciliaires de cas de tuberculose (TB) pulmonaire à frottis positif afin d'évaluer les effets de la mise en œuvre de l'ART sur la contagiosité de la TB.

**MÉTHODE:**

La prévalence de la positivité du test cutané à la tuberculine (TST) a été comparée entre les contacts âgés de 2 à 10 ans de cas index qui étaient VIH négatifs, VIH positifs mais pas sous ART, sous ART depuis moins d'un an et sous ART depuis un an ou plus. Une régression logistique à effets aléatoires a été utilisée afin de tenir compte du regroupement au sein des foyers.

**RÉSULTATS:**

La prévalence de l'infection à M. tuberculosis parmi les contacts des cas index VIH négatifs, VIH positifs sous ART depuis un an ou plus et VIH positifs pas sous ART/sous ART depuis moins d'un an a été de 44%, 21% et 22%, respectivement. Comparés aux contacts des cas index VIH positifs pas sous ART ou seulement mis sous ART récemment, les risques de positivité du TST ont été similaires chez les contacts des cas index VIH positifs sous ART depuis un an ou plus (OR ajusté [ORa] 1,0; IC95% 0,3–3,7). Les risques ont été 2,9 fois plus élevés chez les enfants contacts de cas index VIH négatifs (ORa 2,9 ; IC95% 1,0–8,2).

**CONCLUSION:**

Nous n'avons pas trouvé d'éléments en faveur d'une augmentation de la contagiosité des cas index à frottis positif et VIH positifs à la suite de la mise en route de l'ART.

## Abstract (SP)

**MARCO DE REFERENCIA:**

La comprensión de los efectos de la infección por el virus de la inmunodeficiencia humana (VIH) y el tratamiento antirretrovírico (TAR) en la dinámica de la transmisión de Mycobacterium tuberculosis es aún incompleta. Se emprendió un estudio transversal de los contactos domiciliarios de casos de tuberculosis (TB) pulmonar con baciloscopia positiva, con el fin de examinar el efecto de recibir el TAR, sobre la contagiosidad de la TB.

**MÉTODO:**

Se comparó la prevalencia de positividad de la prueba cutánea de la tuberculina en los contactos de 2 a 10 años de edad de los casos iniciales de TB que eran, ya sea, negativos frente al VIH, positivos frente al VIH y no recibían TAR, positivos y que recibían TAR desde hacía menos de un año o positivos frente al VIH y en TAR desde hacía un año o más. Mediante un modelo de regresión logística de efectos aleatorios se puso de manifiesto la presencia de conglomerados en los domicilios.

**RESULTADOS:**

La prevalencia de infección tuberculosa en los contactos de los casos negativos frente al VIH fue 44%, en los casos positivos frente al VIH que recibían TAR desde hacía un año o más fue 21% y en los casos positivos frente al VIH que no recibían TAR o en TAR durante menos de un año fue 22%. Al comparar los contactos de los casos iniciales positivos frente al VIH que no recibían TAR o que lo habían comenzado recientemente, la posibilidad de obtener un resultado positivo de la prueba tuberculínica fue equivalente a la posibilidad de los contactos de casos positivos frente al VIH que recibían TAR desde hacía un año o más (cociente de posibilidades ajustado [aOR] 1,0; IC95% 0,3 a 3,7). La posibilidad fue 2,9 veces más alta en los niños que eran contactos de casos iniciales de TB negativos frente al VIH (aOR 2,9; IC95% 1,0 a 8,2).

**CONCLUSIÓN:**

No se encontraron pruebas de que el hecho de recibir el TAR aumente la contagiosidad de los casos iniciales de TB con baciloscopia positiva que son positivos frente al VIH.

THE HUMAN IMMUNODEFICIENCY VIRUS (HIV) pandemic continues to challenge global tuberculosis (TB) control,^1^,^2^ yet our understanding of the effects of HIV and antiretroviral treatment (ART) on Mycobacterium tuberculosis transmission dynamics remains limited.^3–7^

In HIV-positive individuals, ART reduces TB incidence across all CD4 cell counts;^8^ nevertheless, despite long-term ART, TB incidence remains higher in HIV-positive than in HIV-negative people in both high and low TB burden settings.^9^,^10^ As life expectancy is greatly extended by ART, the cumulative lifetime risk of TB among HIV-positive people remains very high.^9^ Although HIV-positive TB patients with advanced immunosuppression are less likely to transmit to household contacts than their HIV-negative counterparts,^11–17^ partly due to lower sputum bacillary load,^11^,^16^,^17^ ART may increase the infectiousness of TB by modifying clinical manifestations, making them more similar to those in HIV-negative patients.^18^,^19^

Concerns have been raised that increased life expectancy and possible increased infectiousness due to ART might increase TB incidence at a population level.^20^ This might be negated by reduced HIV transmission;^21^,^22^ however, a rebound in TB incidence, exceeding present pre-ART roll-out levels, is possible if good adherence to ART is not sustained.^23^ Programmatic data from South Africa, Malawi and Zimbabwe have shown a reduction in TB incidence, as inferred from trends in TB case notification, in association with ART scale-up.^24^,^25^ However, short-term reductions in TB incidence may be due to protection from progression to disease rather than a reduction in M. tuberculosis transmission.

We examined the effect of ART on M. tuberculosis transmission by measuring the prevalence of M. tuberculosis infection among child contacts of adult smear-positive TB cases.

## METHODS

### Study setting

Karonga District, northern Malawi, is predominantly rural, with an adult HIV prevalence of 9% and new smear-positive TB incidence of 87 per 100 000 adults per year;^26^ 60% of TB cases are HIV-positive.^26^ The first ART clinic opened in 2005, and by 2012, 16 clinics in the district were certified to initiate and provide ART.^27^

### Study design

A cross-sectional household study of all diagnosed smear-positive TB cases in the district was conducted from January 2013 to April 2015. Households were eligible if a smear-positive case had lived there for at least 2 weeks after the onset of symptoms and before initiation of treatment. Bacteriological, demographic and clinical (including HIV and ART status) data from all patients starting anti-tuberculosis treatment in the district have been collected in a TB case cohort study since 1988 (described elsewhere).^26^,^28^

Households were visited approximately 6 weeks after TB diagnosis of an index case (date of first smear-positive sputum). All children aged 2–10 years residing in the household were included. A tuberculin skin test (TST) was administered and read according to standard international guidelines^29^ using 2 international units of RT23 (Statens Serum Institute, Copenhagen, Denmark), and induration was measured 48–72 h later. A positive TST was defined as induration ⩾10 mm. Children aged <2 years were excluded to minimise misclassification of infection status with false-positive TST due to recent bacille Calmette-Guerin (BCG) vaccination. 30

A questionnaire was completed which included data on demographics, BCG vaccination status, exposure to index case (whether index case was mother, duration of sleeping in same room and of living in the same household while index case was symptomatic), and household characteristics (number of residents, socio-economic indicators including quality of dwelling place). A composite score for household socio-economic status was created using head of household employment, number of assets, food security and availability of soap, and a composite score for quality of dwelling place was based on building materials, type of roof, number of rooms, water source, presence of glass windows, electricity and latrine type.

Any child with symptoms suggestive of TB (fever, weight loss, failure to thrive, night sweats or cough) was reviewed by a clinician and referred to the district hospital where appropriate. All children aged <5 years without evidence of active disease were commenced on 6-month isoniazid preventive treatment (5 mg/kg once daily), irrespective of TST induration size, in accordance with Malawi National TB Programme guidelines.^31^

### Ethics approval

The study was approved by the Malawi National Health Sciences Research Committee, Lilongwe, Malawi (#1049) and the London School of Hygiene & Tropical Medicine Ethics Committee, London, UK (#6285). At the time of study recruitment, smear-positive pulmonary TB patients were asked for written consent to visit their household(s) to screen household members for infection and disease. Written informed consent was then obtained from a parent or guardian of each participating child at the time of household visit.

### Statistical analysis

Prevalence of TST positivity was compared between household contacts by HIV and ART status of the index case to distinguish those not on ART or on ART for <1 year from those on ART for ⩾1 year at TB diagnosis. We performed univariable analyses for covariates known to be risk factors for M. tuberculosis infection, and these were assessed as confounders of the association between HIV/ART status and TST positivity, first individually and then in a multivariable model, using random-effects logistic regression to account for clustering within households.

Sputum smear grade and duration of symptoms were not included in the initial multivariable model as they were considered to be on the causal pathway between HIV/ART status and prevalence of TST positivity in the child contact. These were examined as mediators of the association in subsequent models.

### Sensitivity analyses

The analysis was repeated 1) grouping all patients on ART irrespective of time on ART, 2) separating patients on ART for ⩾2 years and 3) using a TST cutoff ⩾15 mm.

## RESULTS

**Figure 1:**
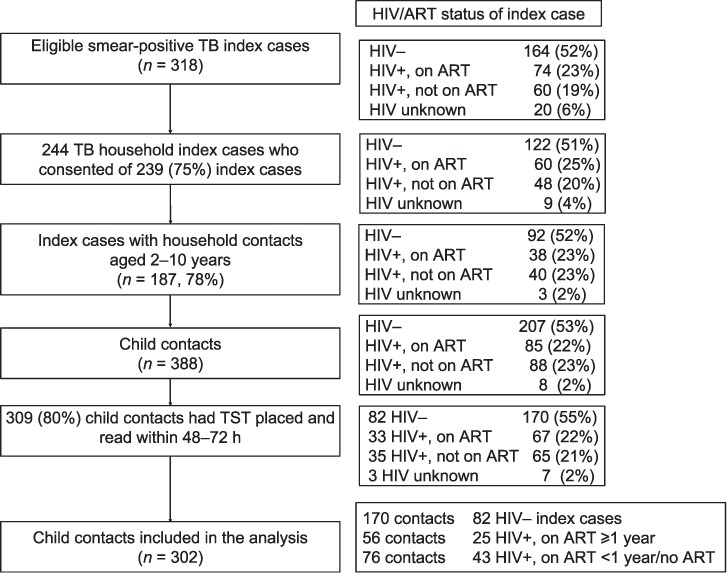
Study flowchart: TB case through to child contact TST data included in analysis. HIV = human immunodeficiency virus; ART =antiretroviral therapy; TB =tuberculosis; −=negative; +=positive.

**Figure 2:**
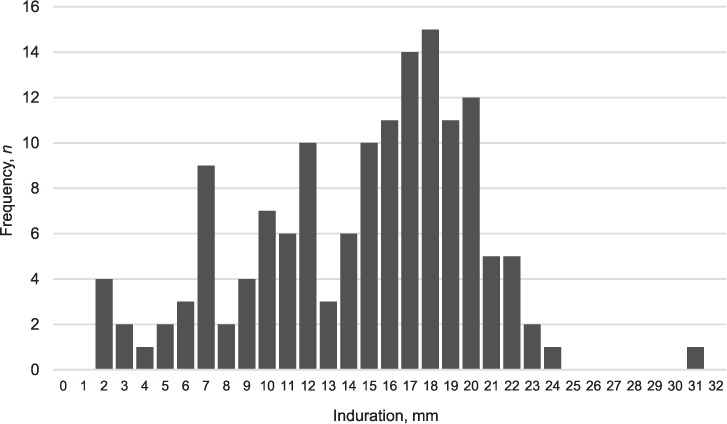
Histogram illustrating the frequency distribution of non-zero induration in child contacts (n = 125).

A total of 388 child contacts of 187 index cases were eligible for inclusion (Figure 1), 309 of whom had a TST placed and read within 48–72 h (80%; 153 index cases). As HIV/ART status was missing for three index cases (seven contacts), 302 child contacts of 150 index cases were included in the final analysis. One hundred and seventy-seven children (58.4%) had no induration; the frequency distribution of those children with non-zero TST induration (*n* = 125) is shown in Figure 2.

### Index case characteristics

Of the 150 index cases, 63% were male. The median age was 33.6 years (interquartile range [IQR] 28.2–39.3) in female index cases and 37.6 years (IQR 30.6–44.9) in male index cases; 22% of index cases were on ARTat TB diagnosis, with a median duration on ART of 2.8 years (IQR 1.2–4.5; *n* = 33). Table 1 shows index case characteristics by HIV/ART status. The median duration of symptoms before TB diagnosis (by self-report) was shortest in HIV-positive patients on ART ⩾1 year (8.3 weeks, IQR 7.0–16.3).

**Table 1:**
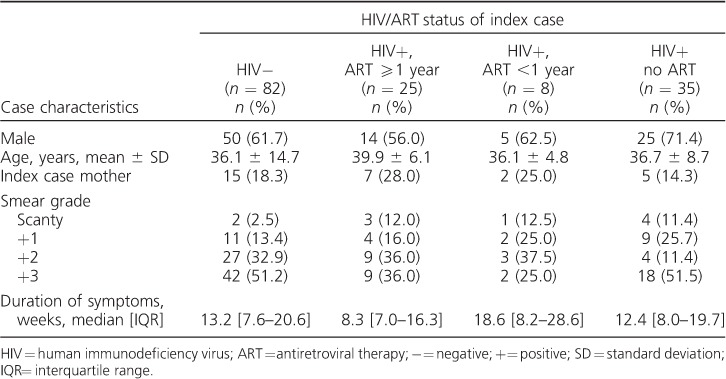
Index case characteristics by HIV/ART status of the index case (n = 150)

### Tuberculin skin test positivity in child contacts

The prevalence of TST positivity among all child contacts was 34.4%. TST positivity in child contacts of HIV-positive index cases not on ARTwas 23.1% (15/65); as there were only 11 child contacts of 8 HIV-positive index cases on ART for <1 year (TST positivity 18.2%), this category was combined with the HIV-positive index cases not on ART. TST positivity was respectively 44.1% (75/170), 21.4% (12/56) and 22.4% (17/76) in contacts of index cases who were HIV-negative, HIV-positive on ART ⩾1 year and HIV-positive not on ART/on ART for <1 year (Table 2).

**Table 2:**
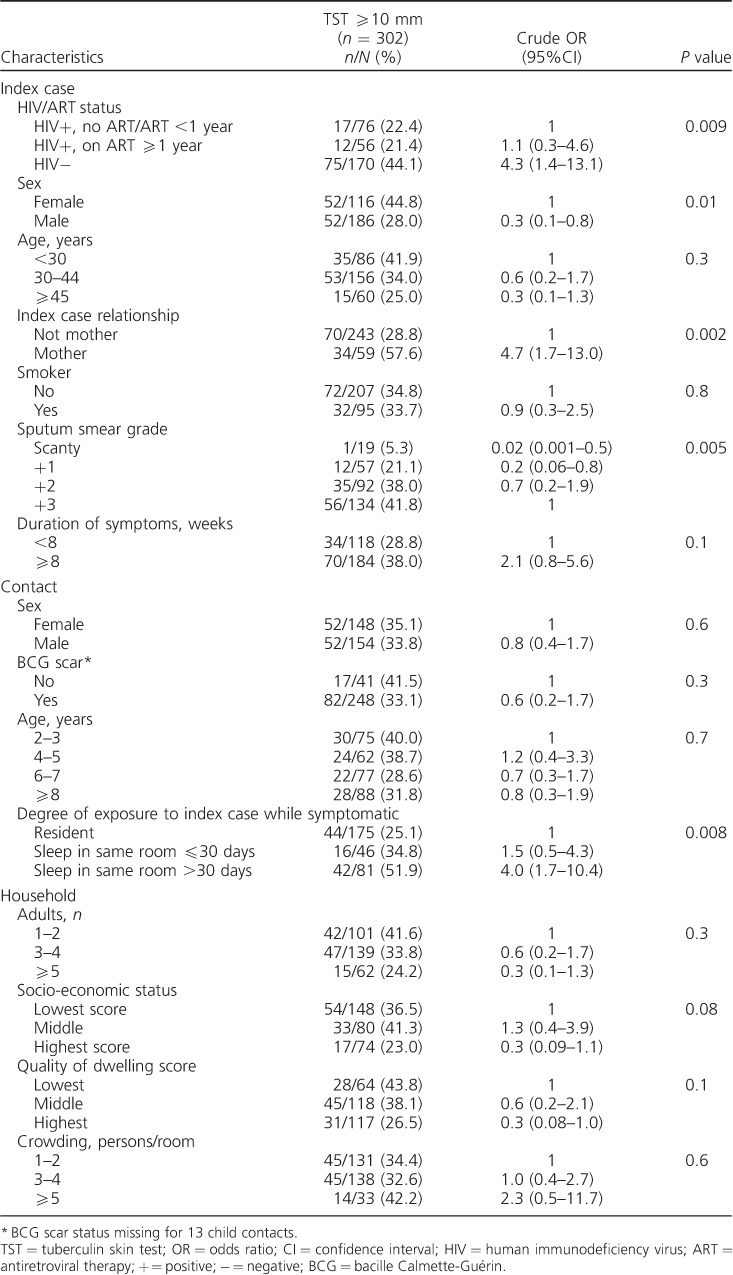
Demographic and clinical characteristics of index case and contact: risk factors for TST positivity (n = 302)

Factors associated with TST positivity in child contacts included HIV/ART status of index case, sex of index case, whether the index case was the mother, index case sputum smear grade and degree of exposure of contact (Table 2).

Compared to contacts of HIV-positive index cases not on ART or on ART for <1 year, the odds of a positive TST were higher in contacts of HIV-negative index cases (crude odds ratio [OR] 4.3, 95%CI 1.4–13.1, reducing to OR 2.9, 95%CI 1.0–8.2 after adjustment for sociodemographic factors), but not in contacts of HIV-positive index cases who had been on ART for ⩾1 year (crude OR 1.1, 95%CI 0.3–4.6, adjusted OR [aOR] 1.0, 95%CI 0.3–3.7; Table 2). Further adjustment was used to assess the effect of factors that may be on the causal pathway. Although adjusting for duration of symptoms (Model 2A, Table 3) made little difference to the odds of TST positivity in contacts of HIV-negative index cases, adjusting for smear grade (Model 2B) reduced the association (aOR 2.2, 95%CI 0.8–6.3). No association of TST positivity with ART duration was observed among contacts of HIV-positive index cases in any model (Table 3).

**Table 3:**
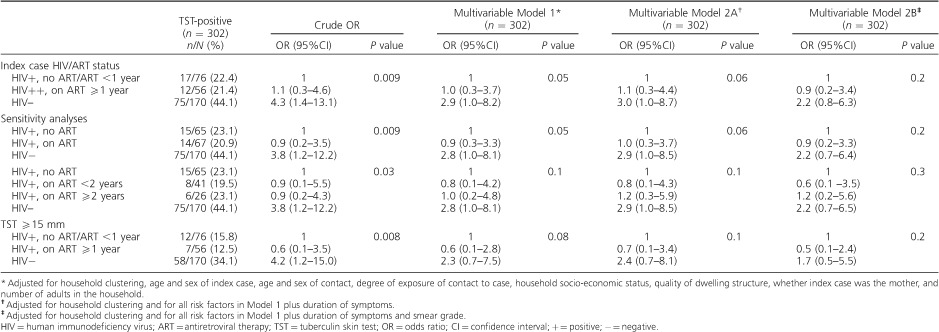
Multivariable analysis of association of HIV/ART status of index case with TST positivity in child contacts

The results of the sensitivity analyses are also shown in Table 3. Using a cut-off TST of ⩾15 mm, regrouping those on ART ignoring duration, or separating those on ART >2 years, made little difference to the results

## DISCUSSION

We found no evidence that child contacts of HIV-positive TB patients on ART were more likely to have a positive TST than child contacts of HIV-positive TB patients not on ART. However, child contacts of HIV-negative individuals had nearly three times the odds of having a positive TST than child contacts of HIV-positive TB patients not on ART; this was partly explained by differences in the degree of smear positivity.

Some TB household contact studies have found no difference in infectiousness between HIV-positive TB patients compared to HIV-negative TB patients if HIV-positive index cases were less immunosuppressed (CD4 >250 cells/mm^3^)^16^ or were smear-positive and/or had cavitary disease.^17^ Heterogeneity observed in estimates of infectiousness of HIV-positive TB patients compared to HIV-negative TB patients has been well-described, although no studies to date have examined the effect of ART status of the HIV-positive index case. Possible reasons for heterogeneity include differences in patient eligibility (smear-positive only vs. all TB patients), study settings (high vs. low HIV and TB background prevalence), household contacts screened for M. tuberculosis infection (adults and children vs. children only) and study-related biases, such as exposure assessment bias and recall bias.^16^,^17^,^32^,^33^ Study calendar period may also influence estimates of infectiousness, as the degree of immunosuppression of HIV-positive TB cases on a population level will be a function of the maturity of the HIV epidemic and the time since roll-out of ART.

The lack of evidence for increased infectiousness in TB patients established on ART compared to those not on ART in our study may be due to earlier diagnosis, resulting in a shorter duration of infectiousness. However, adjusting for duration of symptoms in Model 2A did not alter the odds of a positive TST in contacts of index cases who had been on ART for ⩾1 year. This might be due to the fact that the duration of symptoms at the time of TB diagnosis is notoriously difficult to recall accurately. It should be noted that a contemporaneous study of attendance at the HIV/ART clinic undertaken at the Karonga District Hospital found that HIV-positive individuals on ART attended the clinic much more regularly than those not on ART; the median number of visits per year was 5 (IQR 4–6) among ART patients and 1 (IQR 1–2) among HIV patients not on ART (unpublished data). This gives much greater opportunity for early diagnosis of TB. Another reason for the absence of evidence of increased infectiousness in TB patients established on ART may be that in our north Malawi population the effect of ART was masked by the degree of immunodeficiency of the index case, because ART is started in individuals with more advanced HIV infection.

CD4 cell count testing is not routinely performed in Karonga, resulting in the absence of a biological marker for the level of immunosuppression of HIV-positive TB patients and degree of immune reconstitution in those established on ART in this study. Limitations of our study also include the absence of radiological data to assess the extent of lung cavitation by HIV/ART status. These data would have helped to interpret the apparent lack of effect of ART on infectiousness. Knowledge of the HIV status of the child contacts would also have strengthened this study, as an HIV-positive child with advanced immunosuppression may be more likely to have a false-negative TST than an HIV-negative child, and an HIV-positive child is more likely to be resident in the household of an HIV-positive index case. This is one potential explanation for the lower prevalence of M. tuberculosis infection in child contacts of index cases with HIV infection, irrespective of ART status, found in this study. However, ART for prevention of mother-to-child transmission is widely used and the proportion of infected children will be very low.

## CONCLUSION

We found a higher prevalence of M. tuberculosis infection among child contacts of HIV-negative TB patients than in contacts of HIV-positive index cases, irrespective of ART status. We found no evidence to suggest that HIV-positive index cases on ART for ⩾1 year at TB diagnosis were more likely to transmit than other HIV-positive index cases. The frequent contact of HIV-positive individuals on ART with the health services, leading to prompt diagnosis of TB, may mitigate the effects of any increase in infectiousness. Further studies are required to definitively establish whether ART has a biological effect on the infectiousness of HIV-positive TB patients.

## References

[1] World Health Organization Global tuberculosis control, 2015. WHO/HTM/TB/2015.22 Geneva, Switzerland: WHO, 2015 http://apps.who.int/iris/bitstream/10665/191102/1/9789241565059_eng.pdf?ua=1. Accessed August 2017.

[2] Chaisson R E, Churchyard G J. Recurrent tuberculosis: relapse, reinfection, and HIV. J Infect Dis 2010; 201: 653– 655. 20121432 10.1086/650531 PMC3407677

[3] Yates T A, Khan P Y, Knight G M, The transmission of Mycobacterium tuberculosis in high burden settings. Lancet Infect Dis 2016; 16: 227– 238. 26867464 10.1016/S1473-3099(15)00499-5

[4] Odhiambo J A, Borgdorff M W, Kiambih F M, Tuberculosis and the HIV epidemic: increasing annual risk of tuberculous infection in Kenya, 1986–1996. Am J Public Health 1999; 89: 1078– 1082. 10394319 10.2105/ajph.89.7.1078 PMC1508825

[5] Egwaga S M, Cobelens FG, Muwinge H, Verhage C, Kalisvaart N, Borgdorff M W. The impact of the HIV epidemic on tuberculosis transmission in Tanzania. AIDS 2006; 20: 915– 921. 16549977 10.1097/01.aids.0000218557.44284.83

[6] Middelkoop K, Bekker L G, Myer L, Dawson R, Wood R. Rates of tuberculosis transmission to children and adolescents in a community with a high prevalence of HIV infection among adults. Clin Infect Dis 2008; 47: 349– 355. 18558885 10.1086/589750 PMC3816246

[7] Rieder H L. Editorial commentary: on the risk of being and becoming infected with Mycobacterium tuberculosis. Clin Infect Dis 2008; 47: 356– 357. 18558874 10.1086/589751

[8] Suthar A B, Lawn S D, del Amo J, Antiretroviral therapy for prevention of tuberculosis in adults with HIV: a systematic review and meta-analysis. PLoS Med 2012; 9: e1001270. 22911011 10.1371/journal.pmed.1001270 PMC3404110

[9] Lawn S D, Harries A D, Williams B G, Antiretroviral therapy and the control of HIV-associated tuberculosis. Will ART do it?. Int J Tuberc Lung Dis 2011; 15: 571– 581. 21756508 10.5588/ijtld.10.0483 PMC4067901

[10] Gupta R K, Rice B, Brown A E, Does antiretroviral therapy reduce HIV-associated tuberculosis incidence to background rates? A national observational cohort study from England, Wales, and Northern Ireland. Lancet HIV 2015; 2: e243– 251. 26423197 10.1016/S2352-3018(15)00063-6

[11] Elliott A M, Hayes R J, Halwiindi B, The impact of HIV on infectiousness of pulmonary tuberculosis: a community study in Zambia. AIDS 1993; 7: 981– 987. 8357557 10.1097/00002030-199307000-00012

[12] Cauthen G M, Dooley S W, Onorato I M, Transmission of Mycobacterium tuberculosis from tuberculosis patients with HIV infection or AIDS. Am J Epidemiol 1996; 144: 69– 77. 8659487 10.1093/oxfordjournals.aje.a008856

[13] Espinal M A, Perez E N, Baez J, Infectiousness of Mycobacterium tuberculosis in HIV-1-infected patients with tuberculosis: a prospective study. Lancet 2000; 355: 275– 280. 10675075 10.1016/S0140-6736(99)04402-5

[14] Carvalho A C, DeRiemer K, Nunes Z B, Transmission of Mycobacterium tuberculosis to contacts of HIV-infected tuberculosis patients. Am J Respir Crit Care Med 2001; 164: 2166– 2171. 11751181 10.1164/ajrccm.164.12.2103078

[15] Kenyon T A, Creek T, Laserson K, Risk factors for transmission of Mycobacterium tuberculosis from HIV-infected tuberculosis patients, Botswana. Int J Tuberc Lung Dis 2002; 6: 843– 850. 12365569

[16] Huang C C, Tchetgen E T, Becerra M C, The effect of HIV-related immunosuppression on the risk of tuberculosis transmission to household contacts. Clin Infect Dis 2014; 58: 765– 774. 24368620 10.1093/cid/cit948 PMC3935504

[17] Martinez L, Sekandi J N, Castellanos M E, Zalwango S, Whalen C C. Infectiousness of HIV seropositive tuberculosis patients in a high-burden African setting. Am J Respir Crit Care Med 2016; 194: 1152– 1163. 27181053 10.1164/rccm.201511-2146OC PMC5114446

[18] Munthali L, Khan P Y, Mwaungulu N J, The effect of HIV and antiretroviral therapy on characteristics of pulmonary tuberculosis in northern Malawi: a cross-sectional study. BMC Infect Dis 2014; 14: 107. 24568242 10.1186/1471-2334-14-107 PMC3941771

[19] van Halsema C L, Fielding K L, Chihota V N, Brief report: the effect of antiretroviral therapy and CD4 count on markers of infectiousness in HIV-associated tuberculosis. J Acquir Immune Defic Syndr 2015; 70: 104– 108. 26322671 10.1097/QAI.0000000000000684

[20] Godfrey-Faussett P, Ayles H. Can we control tuberculosis in high HIV prevalence settings? Tuberculosis (Edinb) 2003; 83: 68– 76. 12758192 10.1016/s1472-9792(02)00083-5

[21] Williams B G, Granich R, De Cock K M, Glaziou P, Sharma A, Dye C. Antiretroviral therapy for tuberculosis control in nine African countries. Proc Natl Acad Sci USA 2010; 107: 19485– 19489. 20974976 10.1073/pnas.1005660107 PMC2984151

[22] Pretorius C, Menzies N A, Chindelevitch L, The potential effects of changing HIV treatment policy on tuberculosis outcomes in South Africa: results from three tuberculosis-HIV transmission models. AIDS 2014; 28 Suppl 1: S25– S34. 24468944 10.1097/QAD.0000000000000085

[23] Dodd P J, Knight G M, Lawn S D, Corbett E L, White R G. Predicting the long-term impact of antiretroviral therapy scale-up on population incidence of tuberculosis. PLoS ONE 2013; 8: e75466. 24069418 10.1371/journal.pone.0075466 PMC3775764

[24] Zachariah R, Bemelmans M, Akesson A, Reduced tuberculosis case notification associated with scaling up antiretroviral treatment in rural Malawi. Int J Tuberc Lung Dis 2011; 15: 933– 937. 21682967 10.5588/ijtld.10.0666

[25] Middelkoop K, Wood R, Bekker L G. The impact of antiretroviral treatment programs on tuberculosis notification rates. Int J Tuberc Lung Dis 2011; 15: 1714; author reply 1714–1715. 22118185 10.5588/ijtld.11.0545

[26] Mboma S M, Houben R M, Glynn J R, Control of (multi)drug resistance and tuberculosis incidence over 23 years in the context of a well-supported tuberculosis programme in Rural Malawi. PLoS ONE 2013; 8: e58192. 23483994 10.1371/journal.pone.0058192 PMC3590148

[27] Koole O, Houben R M, Mzembe T, Improved retention of patients starting antiretroviral treatment in Karonga District, northern Malawi, 2005–2012. J Acquir Immune Defic Syndr 2014; 67: e27– 33. 24977375 10.1097/QAI.0000000000000252 PMC4240943

[28] Crampin A C, Glynn J R, Fine P E. What has Karonga taught us? Tuberculosis studied over three decades. Int J Tuberc Lung Dis 2009; 13: 153– 164. 19146741 PMC3272402

[29] Arnadottir T, Rieder H L, Trébucq A, Waaler H T. Guidelines for conducting tuberculin skin test surveys in high prevalence countries. Tubercle Lung Dis 1996; 77 Suppl 1: 1– 19. 10.1016/s0962-8479(96)90127-6 8759471

[30] Khan P Y, Glynn J R, Fielding K L, Risk factors for Mycobacterium tuberculosis infection in 2–4 year olds in a rural HIV-prevalent setting. Int J Tuberc Lung Dis 2016; 20: 342– 349. 27046715 10.5588/ijtld.15.0672 PMC4743681

[31] Ministry of Health Malawi National Tuberculosis Control Programme Manual. Lilongwe, Malawi: Ministry of Health, 2007.

[32] Cruciani M, Malena M, Bosco O, Gatti G, Serpelloni G. The impact of human immunodeficiency virus type 1 on infectiousness of tuberculosis: a meta-analysis. Clin Infect Dis 2001; 33: 1922– 1930. 11692305 10.1086/324352

[33] Pai M, McCulloch M, Colford J M Jr. Meta-analysis of the impact of HIV on the infectiousness of tuberculosis: methodological concerns. Clin Infect Dis 2002; 34: 1285– 1287. 11941565 10.1086/339951

